# Diaqua­bis­(1*H*-imidazole-κ*N* ^3^)bis­(4-nitro­benzoato-κ*O* ^1^)cadmium

**DOI:** 10.1107/S1600536812033739

**Published:** 2012-08-04

**Authors:** Yan-Li Mao, Xiao-Ke Yu, Jian-Li Lin

**Affiliations:** a Center of Applied Solid State Chemistry Research, Ningbo University, Ningbo, Zhejiang 315211, People’s Republic of China

## Abstract

In the centrosymmetric title compound, [Cd(C_7_H_4_NO_4_)_2_(C_3_H_4_N_2_)_2_(H_2_O)_2_], the Cd^II^ atom, located on an inversion center, is coordinated by two N atoms and four O atoms in an octa­hedral geometry. The inter­nal cohesion of the mol­ecule is enhanced by an intra­molecular O—H⋯O hydrogen bond. Inter­molecular O—H⋯O and C—H⋯O hydrogen bonds and π–π contacts [centroid–centroid distance = 3.6549 (2) Å] define two-dimensional networks parallel to (001), which are further connected by weaker C—H⋯O inter­actions into a weakly connected three-dimensional supra­molecular framework.

## Related literature

For general background to aromatic carboxyl acid complexes, see: Kuang *et al.* (2007 ▶); Hsu *et al.* (2011 ▶). For related structures, see: Zheng *et al.* (2008 ▶).
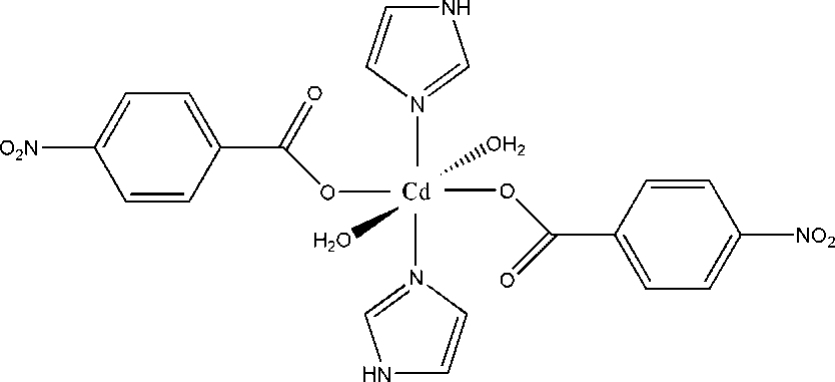


## Experimental

### 

#### Crystal data

[Cd(C_7_H_4_NO_4_)_2_(C_3_H_4_N_2_)_2_(H_2_O)_2_]
*M* *_r_* = 614.80
Triclinic, 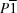

*a* = 5.8017 (12) Å
*b* = 8.0253 (16) Å
*c* = 12.879 (3) Å
α = 77.99 (3)°
β = 88.42 (3)°
γ = 85.16 (3)°
*V* = 584.4 (2) Å^3^
*Z* = 1
Mo *K*α radiation
μ = 1.00 mm^−1^
*T* = 293 K
0.33 × 0.14 × 0.09 mm

#### Data collection

Rigaku R-AXIS RAPID diffractometer
Absorption correction: multi-scan (*ABSCOR*; Higashi, 1995 ▶) *T*_min_ = 0.989, *T*_max_ = 0.989
5719 measured reflections
2627 independent reflections
2511 reflections with *I* > 2σ(*I*)
*R*_int_ = 0.031

#### Refinement

*R*[*F* ^2^ > 2σ(*F* ^2^)] = 0.026
*wR*(*F* ^2^) = 0.076
*S* = 1.24
2627 reflections
175 parameters
3 restraints
H atoms treated by a mixture of independent and constrained refinement
Δρ_max_ = 0.54 e Å^−3^
Δρ_min_ = −0.80 e Å^−3^

### 

Data collection: *RAPID-AUTO* (Rigaku, 1998 ▶); cell refinement: *RAPID-AUTO*; data reduction: *CrystalStructure* (Rigaku/MSC, 2004 ▶); program(s) used to solve structure: *SHELXS97* (Sheldrick, 2008 ▶); program(s) used to refine structure: *SHELXL97* (Sheldrick, 2008 ▶); molecular graphics: *SHELXTL* (Sheldrick, 2008 ▶); software used to prepare material for publication: *SHELXL97*.

## Supplementary Material

Crystal structure: contains datablock(s) global, I. DOI: 10.1107/S1600536812033739/bg2473sup1.cif

Structure factors: contains datablock(s) I. DOI: 10.1107/S1600536812033739/bg2473Isup2.hkl

Additional supplementary materials:  crystallographic information; 3D view; checkCIF report

## Figures and Tables

**Table 1:** Hydrogen-bond geometry (Å, °)

*D*—H⋯*A*	*D*—H	H⋯*A*	*D*⋯*A*	*D*—H⋯*A*
O5—H5*A*⋯O2	0.84 (1)	1.88 (1)	2.679 (1)	159
O5—H5*B*⋯O1^i^	0.84 (1)	1.97 (1)	2.785 (1)	164
C2—H2*A*⋯O5^ii^	0.93	2.58	3.244 (1)	129
C3—H3*A*⋯O5^iii^	0.93	2.43	3.344 (1)	169
C10—H10*A*⋯O2	0.93	2.42	2.751 (4)	101

Symmetry codes: (i) 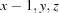; (ii) 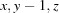; (iii) 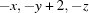.

**Fig. 1.**
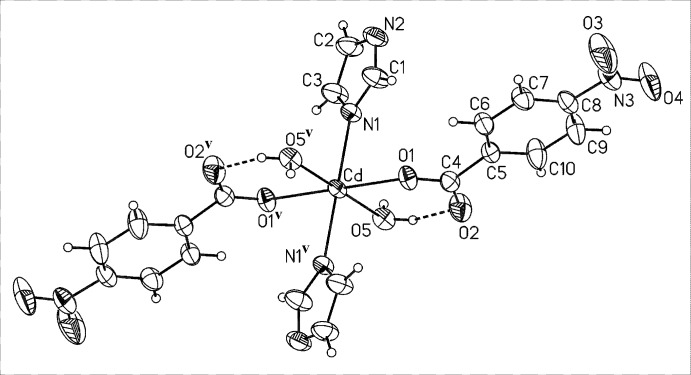
Figure 1. ORTEP view of the title compound, The dispalcement ellipsoidsare drawn at 45% probability dispalcement ellipsoids. Symmetry code: (v)1-x, 2-y, -z.

**Fig. 2.**
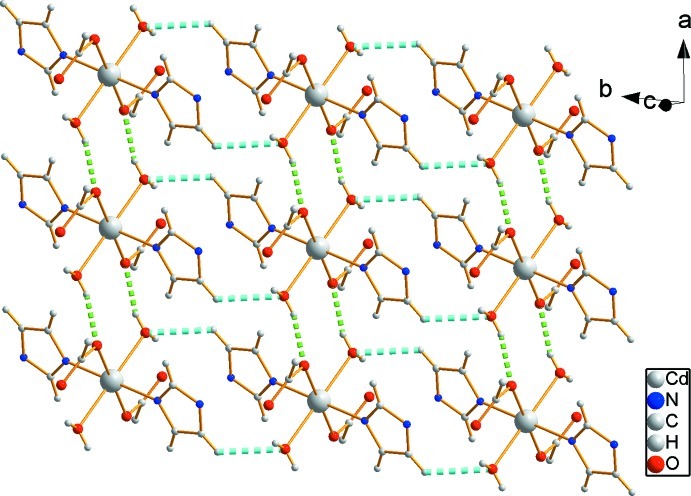
The two-dimensional supramolecular networks parallel to (001). In order to observe how the complex moleculars form two-dimensional layers clearly, nitrobenzene on 4-nitrobenzoato molecules which are not engaged in link the components into a two-dimensional layers were omitted.

**Fig. 3.**
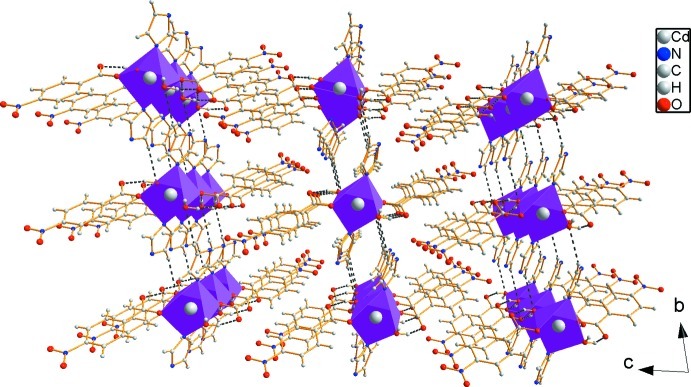
The three-dimensional framework of title complex.

## supplementary crystallographic information

### Comment

Aromatic carboxyl acid complexes have been paid great attention these years for their potential applications in gas storage, separation, catalysis, magnetism, luminescence, and drug delivery (Kuang *et al.*, 2007). As a N-containing aromatic carboxyl acid, nitrobenzoic acid has been widely used in dye intermediate, organic synthesis, sensitization material, functional pigment (Hsu *et al.*, 2011). So far, to our knowledge, cadmium complexes constructed from 4-nitrobenzoato and imidazole have not been reported. In order to get new Cd^II^ complexes with novel functions and discover their structure-property relationship, a new complex [Cd(C_7_H_4_NO_4_)(C_3_H_4_N_2_)(H_2_O)] was synthesized.

The asymmetric unit of [Cd(C_7_H_4_NO_4_)(C_3_H_4_N_2_)(H_2_O)] consists of a Cd^2+^ ion lying on an inversion centre, a 4-NBA^-^ ion (4-HNBA = 4-nitrobenzoic acid), one imidazole ligand and one lattice water as illustrated in Fig. 1. The Cd^2+^ cation is octahedrally coordinated by two N atoms of imidazole ligands, two O atoms from two 4-NBA^-^ ions and two O atoms from two lattice water molecules; it takes a (4 + 2) octahedral geometry, with the oxygen atoms located in the equatorial plane (Cd—O1 = 2.364 (2) Å, Cd—O5 = 2.367 (2) Å, and the two nitrogen atoms occupying the axial position (Cd—N1 = 2.255 (2) Å). Table 1 presents the \p···\p contact information involving the C_3_N_2_ ring (centroid, Cg1) and Table 2, the more meaningful H-bonds in the structure; the most important ones are those involving water H's. The one described in the first entry in Table 2 is intramolecular; the seocnd one, instead defines chains along a (Figure 2, vertical arrays). The weak one involving C2—H2A (Table 2, third entry) and the π–π contact (Table 1) link chains into a two-dimensional supramolecular network parallel to (001) as illustrated in Figure 2. Finaly, the remaining weak H-bonds link these 2D structures into a 3D supramolecular architecture (Figure 3).

### Experimental

Dropwise addition of 1.0 ml (1 *M*) of K_2_CO_3_ to a stirred aqueous solution of Cd(CH_3_COO)_2_.2H_2_O (0.266 g, 1.0 mmol) in 10.0 ml of H_2_O yielded a fine white precipitate, which was separated by centrifugation and washed with water until no CH_3_COO^-^ anions were detectable in the supernatant. The fresh precipitate was then added to a stirred aqueous solution of 4-nitrobenzoic acid (0.167 g, 1.0 mmol) in C_2_H_5_OH/H_2_O (1:1, 20.0 ml), producing a white suspension, to which imidazole (0.137 g, 2.0 mmol) was added. The mixture was further stirred vigorously for about 0.5 h. After filtration, the white filtrate (pH = 6.59) was maintained at room temperature and colorless crystals were grown.

### Refinement

H atoms bonded to C atoms were palced in geometrically calculated positions and were refined using a riding model. H atoms attached to O atoms were found in a difference Fourier synthesis and were refined with restrained O—H = 0.84 (1)Å. In all cases, *U*_iso_(H) values were set at 1.2 *U*eq(host).

### Crystal data

**Table:**

[Cd(C_7_H_4_NO_4_)_2_(C_3_H_4_N_2_)_2_(H_2_O)_2_]	*Z* = 1
*M**_r_* = 614.80	*F*(000) = 308
Triclinic, *P*1	*D*_x_ = 1.747 Mg m^−^^3^
Hall symbol: -P 1	Mo *K*α radiation, λ = 0.71073 Å
*a* = 5.8017 (12) Å	Cell parameters from 5719 reflections
*b* = 8.0253 (16) Å	θ = 3.2–27.5°
*c* = 12.879 (3) Å	µ = 1.00 mm^−^^1^
α = 77.99 (3)°	*T* = 293 K
β = 88.42 (3)°	Plate, colorless
γ = 85.16 (3)°	0.33 × 0.14 × 0.09 mm
*V* = 584.4 (2) Å^3^	

### Data collection

**Table:**

Rigaku R-AXIS RAPID diffractometer	2627 independent reflections
Radiation source: fine-focus sealed tube	2511 reflections with *I* > 2σ(*I*)
Graphite monochromator	*R*_int_ = 0.031
ω scans	θ_max_ = 27.5°, θ_min_ = 3.2°
Absorption correction: multi-scan (*ABSCOR*; Higashi, 1995)	*h* = −6→7
*T*_min_ = 0.989, *T*_max_ = 0.989	*k* = −10→10
5719 measured reflections	*l* = −16→16

### Refinement

**Table:**

Refinement on *F*^2^	Primary atom site location: structure-invariant direct methods
Least-squares matrix: full	Secondary atom site location: difference Fourier map
*R*[*F*^2^ > 2σ(*F*^2^)] = 0.026	Hydrogen site location: inferred from neighbouring sites
*wR*(*F*^2^) = 0.076	H atoms treated by a mixture of independent and constrained refinement
*S* = 1.24	*w* = 1/[σ^2^(*F*_o_^2^) + (0.0208*P*)^2^ + 0.3927*P*] where *P* = (*F*_o_^2^ + 2*F*_c_^2^)/3
2627 reflections	(Δ/σ)_max_ < 0.001
175 parameters	Δρ_max_ = 0.54 e Å^−^^3^
3 restraints	Δρ_min_ = −0.80 e Å^−^^3^

### Special details

**Table:**

Geometry. All esds (except the esd in the dihedral angle between two l.s. planes) are estimated using the full covariance matrix. The cell esds are taken into account individually in the estimation of esds in distances, angles and torsion angles; correlations between esds in cell parameters are only used when they are defined by crystal symmetry. An approximate (isotropic) treatment of cell esds is used for estimating esds involving l.s. planes.

### Fractional atomic coordinates and isotropic or equivalent isotropic displacement parameters (Å^2^)

**Table:**

	*x*	*y*	*z*	*U*_iso_*/*U*_eq_	
Cd	0.5000	1.0000	0.0000	0.03040 (10)	
N1	0.4161 (4)	0.7310 (3)	0.07261 (18)	0.0351 (5)	
C1	0.5412 (6)	0.6131 (4)	0.1376 (3)	0.0497 (8)	
H1A	0.6844	0.6290	0.1632	0.060*	
C2	0.2360 (6)	0.4946 (4)	0.1116 (3)	0.0518 (8)	
H2A	0.1261	0.4160	0.1136	0.062*	
N2	0.4388 (5)	0.4676 (3)	0.1627 (2)	0.0500 (7)	
C3	0.2227 (6)	0.6573 (4)	0.0569 (3)	0.0510 (8)	
H3A	0.0992	0.7108	0.0147	0.061*	
O1	0.7532 (3)	0.9739 (3)	0.14420 (15)	0.0384 (5)	
O2	0.4950 (4)	1.0755 (3)	0.25118 (18)	0.0553 (6)	
C4	0.6746 (5)	0.9881 (4)	0.2347 (2)	0.0336 (6)	
C5	0.8110 (5)	0.8918 (4)	0.3301 (2)	0.0343 (6)	
C6	1.0191 (5)	0.7974 (4)	0.3212 (2)	0.0398 (7)	
H6A	1.0781	0.7893	0.2544	0.048*	
C7	1.1397 (5)	0.7150 (4)	0.4110 (2)	0.0451 (7)	
H7A	1.2798	0.6518	0.4054	0.054*	
C8	1.0480 (5)	0.7285 (4)	0.5088 (2)	0.0421 (7)	
C9	0.8406 (6)	0.8178 (5)	0.5205 (3)	0.0581 (10)	
H9A	0.7807	0.8235	0.5875	0.070*	
C10	0.7234 (6)	0.8990 (5)	0.4301 (3)	0.0568 (9)	
H10A	0.5821	0.9601	0.4365	0.068*	
N3	1.1799 (5)	0.6471 (4)	0.6046 (2)	0.0548 (7)	
O3	1.3619 (6)	0.5690 (5)	0.5945 (3)	0.1018 (13)	
O4	1.1007 (6)	0.6639 (5)	0.6907 (2)	0.0890 (10)	
O5	0.1847 (3)	1.1006 (3)	0.09734 (16)	0.0385 (5)	
H5A	0.252 (5)	1.088 (4)	0.1559 (14)	0.046*	
H5B	0.069 (4)	1.043 (4)	0.112 (2)	0.046*	

### Atomic displacement parameters (Å^2^)

**Table:**

	*U*^11^	*U*^22^	*U*^33^	*U*^12^	*U*^13^	*U*^23^
Cd	0.03111 (16)	0.02902 (15)	0.02818 (15)	−0.00363 (11)	−0.00305 (10)	0.00151 (10)
N1	0.0371 (12)	0.0309 (11)	0.0343 (12)	−0.0032 (10)	−0.0033 (10)	0.0009 (9)
C1	0.0417 (17)	0.0403 (16)	0.060 (2)	−0.0052 (14)	−0.0155 (15)	0.0081 (14)
C2	0.057 (2)	0.0329 (15)	0.063 (2)	−0.0156 (15)	−0.0132 (17)	0.0023 (14)
N2	0.0605 (17)	0.0342 (13)	0.0488 (16)	−0.0008 (13)	−0.0064 (13)	0.0059 (11)
C3	0.0523 (19)	0.0360 (15)	0.061 (2)	−0.0116 (15)	−0.0226 (16)	0.0051 (14)
O1	0.0343 (10)	0.0508 (12)	0.0283 (10)	−0.0078 (9)	−0.0042 (8)	−0.0022 (8)
O2	0.0432 (12)	0.0741 (16)	0.0449 (13)	0.0183 (12)	−0.0127 (10)	−0.0115 (11)
C4	0.0287 (13)	0.0375 (14)	0.0339 (14)	−0.0045 (12)	−0.0064 (11)	−0.0045 (11)
C5	0.0330 (14)	0.0394 (14)	0.0291 (14)	−0.0040 (12)	−0.0038 (11)	−0.0031 (11)
C6	0.0398 (15)	0.0456 (16)	0.0306 (14)	0.0047 (13)	−0.0003 (12)	−0.0034 (12)
C7	0.0398 (16)	0.0488 (17)	0.0413 (17)	0.0117 (14)	−0.0032 (13)	−0.0023 (13)
C8	0.0430 (16)	0.0457 (16)	0.0331 (15)	−0.0038 (14)	−0.0107 (12)	0.0036 (12)
C9	0.052 (2)	0.090 (3)	0.0283 (16)	0.0104 (19)	0.0000 (14)	−0.0078 (16)
C10	0.0446 (18)	0.087 (3)	0.0346 (17)	0.0216 (18)	−0.0033 (14)	−0.0120 (16)
N3	0.0543 (18)	0.0620 (18)	0.0407 (16)	−0.0015 (15)	−0.0138 (13)	0.0067 (13)
O3	0.079 (2)	0.147 (3)	0.0583 (19)	0.052 (2)	−0.0207 (16)	0.0044 (19)
O4	0.090 (2)	0.131 (3)	0.0339 (15)	0.017 (2)	−0.0135 (14)	0.0035 (16)
O5	0.0283 (10)	0.0424 (11)	0.0415 (12)	−0.0039 (9)	−0.0024 (8)	−0.0005 (9)

### Geometric parameters (Å, º)

**Table:**

Cd—N1^i^	2.254 (2)	C4—C5	1.513 (4)
Cd—N1	2.254 (2)	C5—C10	1.383 (4)
Cd—O1^i^	2.364 (2)	C5—C6	1.385 (4)
Cd—O1	2.364 (2)	C6—C7	1.383 (4)
Cd—O5^i^	2.370 (2)	C6—H6A	0.9300
Cd—O5	2.370 (2)	C7—C8	1.375 (4)
N1—C1	1.308 (4)	C7—H7A	0.9300
N1—C3	1.351 (4)	C8—C9	1.369 (5)
C1—N2	1.330 (4)	C8—N3	1.471 (4)
C1—H1A	0.9300	C9—C10	1.377 (5)
C2—N2	1.343 (4)	C9—H9A	0.9300
C2—C3	1.345 (4)	C10—H10A	0.9300
C2—H2A	0.9300	N3—O3	1.199 (4)
C3—H3A	0.9300	N3—O4	1.218 (4)
O1—C4	1.263 (3)	O5—H5A	0.842 (10)
O2—C4	1.245 (4)	O5—H5B	0.842 (10)
			
Cg1···Cg1^ii^	3.6549 (2)		
			
N1^i^—Cd—N1	180.0	C4—O1—Cd	120.39 (17)
N1^i^—Cd—O1^i^	86.19 (8)	O2—C4—O1	125.1 (3)
N1—Cd—O1^i^	93.81 (8)	O2—C4—C5	117.7 (3)
N1^i^—Cd—O1	93.81 (8)	O1—C4—C5	117.2 (2)
N1—Cd—O1	86.19 (8)	C10—C5—C6	118.9 (3)
O1^i^—Cd—O1	180.0	C10—C5—C4	118.3 (3)
N1^i^—Cd—O5^i^	88.39 (8)	C6—C5—C4	122.8 (3)
N1—Cd—O5^i^	91.61 (8)	C7—C6—C5	120.5 (3)
O1^i^—Cd—O5^i^	91.85 (7)	C7—C6—H6A	119.8
O1—Cd—O5^i^	88.15 (7)	C5—C6—H6A	119.8
N1^i^—Cd—O5	91.61 (8)	C8—C7—C6	118.5 (3)
N1—Cd—O5	88.39 (8)	C8—C7—H7A	120.7
O1^i^—Cd—O5	88.15 (7)	C6—C7—H7A	120.7
O1—Cd—O5	91.85 (7)	C9—C8—C7	122.5 (3)
O5^i^—Cd—O5	180.0	C9—C8—N3	118.7 (3)
C1—N1—C3	105.2 (2)	C7—C8—N3	118.8 (3)
C1—N1—Cd	128.3 (2)	C8—C9—C10	118.0 (3)
C3—N1—Cd	126.52 (19)	C8—C9—H9A	121.0
N1—C1—N2	111.8 (3)	C10—C9—H9A	121.0
N1—C1—H1A	124.1	C9—C10—C5	121.5 (3)
N2—C1—H1A	124.1	C9—C10—H10A	119.2
N2—C2—C3	106.7 (3)	C5—C10—H10A	119.2
N2—C2—H2A	126.6	O3—N3—O4	122.8 (3)
C3—C2—H2A	126.6	O3—N3—C8	118.8 (3)
C1—N2—C2	106.7 (3)	O4—N3—C8	118.4 (3)
C2—C3—N1	109.6 (3)	Cd—O5—H5A	98 (2)
C2—C3—H3A	125.2	Cd—O5—H5B	120 (2)
N1—C3—H3A	125.2	H5A—O5—H5B	104 (2)

Symmetry codes: (i) −*x*+1, −*y*+2, −*z*; (ii) −*x*+1, −*y*+1, −*z*.

### Hydrogen-bond geometry (Å, º)

**Table:**

*D*—H···*A*	*D*—H	H···*A*	*D*···*A*	*D*—H···*A*
O5—H5*A*···O2	0.84 (1)	1.88 (1)	2.679 (1)	159
O5—H5*B*···O1^iii^	0.84 (1)	1.97 (1)	2.785 (1)	164
C2—H2*A*···O5^iv^	0.93	2.58	3.244 (1)	129
C3—H3*A*···O5^v^	0.93	2.43	3.344 (1)	169
C10—H10*A*···O2	0.93	2.42	2.751 (4)	101

Symmetry codes: (iii) *x*−1, *y*, *z*; (iv) *x*, *y*−1, *z*; (v) −*x*, −*y*+2, −*z*.
